# Residual endogenous corticosteroid production in patients with adrenal insufficiency

**DOI:** 10.1111/cen.14006

**Published:** 2019-06-20

**Authors:** Annet Vulto, Ragnhildur Bergthorsdottir, Martijn van Faassen, Ido P. Kema, Gudmundur Johannsson, André P. van Beek

**Affiliations:** ^1^ Department of Endocrinology University of Groningen, University Medical Center Groningen Groningen The Netherlands; ^2^ Department of Endocrinology Sahlgrenska University Hospital Gothenburg Sweden; ^3^ Department of Internal Medicine and Clinical Nutrition, Institute of Medicine Sahlgrenska Academy, University of Gothenburg Gothenburg Sweden; ^4^ Department of Laboratory Medicine University of Groningen, University Medical Center Groningen Groningen The Netherlands

**Keywords:** 11‐deoxycortisol, adrenal insufficiency, aldosterone, corticosterone, cortisol, hydrocortisone, precursors

## Abstract

**Objective:**

This study aimed at comparing precursors of endogenous corticosteroid production in patients with primary adrenal insufficiency and in secondary adrenal insufficiency.

**Design:**

Twenty patients with primary adrenal insufficiency and matched controls and 19 patients with secondary adrenal insufficiency participated in this ancillary analysis of two different studies.

**Patients and measurements:**

Patients with primary adrenal insufficiency were on stable hydrocortisone and fludrocortisone therapy. Patients with secondary adrenal insufficiency received two different doses of hydrocortisone in a randomized crossover study. Main outcome measures were concentrations of precursors of cortisol and aldosterone measured by LC‐MS/MS

**Results:**

Compared to controls, progressively lower concentrations of the glucocorticoid precursors 11‐deoxycortisol, 11‐deoxycorticosterone and corticosterone concentrations were found in patients with secondary adrenal insufficiency on lower hydrocortisone dose, secondary adrenal insufficiency on higher hydrocortisone dose and primary adrenal insufficiency, respectively. Half of the primary adrenal insufficient patients showed evidence of residual endogenous cortisol or aldosterone synthesis, as determined by quantifiable 11‐deoxycortisol, 11‐deoxycorticosterone and corticosterone conce

ntrations. In secondary adrenal insufficient patients with higher endogenous cortisol production, as indicated by 11‐deoxycortisol concentrations above the median, no increased cortisol exposure was observed both by plasma pharmacokinetic parameters and 24‐hour free cortisol excretion in urine.

**Conclusions:**

Adrenal corticosteroid production is likely to continue during treatment in a considerable percentage of patients with both primary and secondary adrenal insufficiency. In patients with secondary adrenal insufficiency, this synthesis appears to be sensitive to the dose of hydrocortisone. However, the residual corticosteroid concentrations were quantitatively low and its clinical significance remains therefore to be determined.

## 1 INTRODUCTION

Patients with primary adrenal insufficiency (PAI) due to autoimmune adrenalitis are thought to have no adrenal cortical function after long‐standing disease as a consequence of progressive destruction of the adrenal gland. This notion comes from autopsy reports in which the adrenal glands were found to be small after long‐standing disease, with a capsule that was thickened and fibrotic and a cortex that was completely destroyed, although in some cases a few small clusters of adrenocortical cells were found surrounded by lymphocytes.^1^ The previous research has shown that the development of adrenal insufficiency progresses through different stages whereby adrenocortical autoantibodies correlate with the degree of adrenal dysfunction.^2^, ^3^ This suggests that adrenal insufficiency is always the inevitable end result, but interestingly some cases of (partial) recovery of adrenal function in a patient with autoimmune Addison's disease have been described.^4^, ^5^, ^6^, ^7^ Further, in a cross‐sectional study by Smans and Zelissen performed among 27 patients with long‐standing autoimmune Addison's disease 10 patients were found to have detectable cortisol at the end of synacthen testing.^8^ Although none of them showed (partial) recovery of adrenal function, their findings clearly indicate that in one third of patients with long‐standing disease, some residual adrenal function was still present. Even though very rare, testing for residual function may have far reaching consequences because Gan and co‐workers found that the treatment with tetracosactide improved adrenal function in one patient.^9^

Patients with secondary adrenal insufficiency (SAI) have an insufficient production of ACTH due to pituitary injury. The adrenal glands therefore lack the stimulus for adequate cortisol production and become atrophic. At the time of the diagnosis of both PAI and SAI, the early morning cortisol or the stimulated cortisol is very low, but often still measurable. The clinical significance of this residual production is unknown. It can be speculated to reduce the requirements of hormone replacement, affect quality of life, or influence hospital admission rates for Addisonian crisis and even mortality. Once the diagnosis of PAI or SAI is established, patients are rapidly replaced to compensate for the loss of hormone production. Patients suffering from PAI and SAI require cortisol replacement, commonly given as hydrocortisone (HC) tablets. In addition, patients with PAI need fludrocortisone replacement as substitute for aldosterone. These therapies are lifelong. Interestingly, some investigators found that recovery of the adrenal function was not uncommon in secondary adrenal insufficiency even after a median of nearly 2 years.^10^

Few studies have addressed the residual endogenous cortisol production in long‐standing adrenal insufficiency during substitution therapy. This is difficult because the chemical composition of hydrocortisone tablets and endogenous cortisol is identical, and therefore, usually the strategy is chosen to temporarily withdraw hydrocortisone substitution. Alternatively, this problem can be circumvented by using stable isotope dilution/mass spectrometry, but these techniques are difficult and only a few research centres are equipped to do such studies.^11^, ^12^ A candidate approach to determine residual endogenous cortisol or aldosterone production may be the measurement of precursors of these corticosteroids. To explore this option, we recently developed a LC‐MS/MS assay capable of measuring not only cortisol and cortisone in one run but also several precursor corticosteroids including 11‐deoxycortisol (11S), 11‐deoxycorticosterone (11DOC) and corticosterone (B) concentrations. This creates the possibility to routinely map the underlying function of the adrenal cortex in patients on hydrocortisone substitution and to gain further insight in its clinical consequence. The aim of this study was to assess the applicability of these techniques in estimating the residual function of the adrenal cortex in patients with adrenal insufficiency.

## 2 METHODS

### 2.1 Study design

In this study, we compared patients with primary adrenal insufficiency and their controls from a cross‐sectional, single‐centre, case‐control study with patients with secondary adrenal insufficiency from a randomized double‐blind crossover study.

### 2.2 Ethical considerations

In both studies, ethical approval was obtained from the local ethics committee. The study was approved by the University Medical Center Groningen, the Netherlands and Sahlgrenska University Hospital, Gothenburg, Sweden. Informed consent was obtained from all participants before any elements of the study were performed. The study was conducted according to the latest Declaration of Helsinki.

### 2.3 Subjects

#### 2.3.1 Patients with primary adrenal insufficiency and matched controls

Twenty patients and their controls were derived from a larger sample participating in a cross‐sectional, single‐centre, case‐control study conducted at the Centre for Endocrinology and Metabolism, Sahlgrenska University Hospital, Gothenburg, Sweden. The study design and recruitment have been published previously.^13^ In short, adult patients from western Sweden, diagnosed with primary adrenal insufficiency at the age of 18 years or older, were invited to participate. Matched control subjects from the Gothenburg region were recruited from a random population sample obtained from the Swedish Population Registry. The patients took their usual HC morning dose at home before arriving to the hospital. Fasting blood samples were collected in the morning (between 8:00 and 10:00 AM) after an overnight fast.

#### 2.3.2 Patients with secondary adrenal insufficiency

This randomized double‐blind crossover study was performed in a university hospital in Groningen, The Netherlands, between May 2012 and June 2013. The study has been registered with ClinicalTrials.gov with number NCT01546922. The study design and rationale have been published in detail previously.^14^ In short, patients with established secondary adrenal insufficiency (based on internationally accepted criteria) were recruited from the endocrine outpatient clinic at the University Medical Center Groningen, The Netherlands. The following inclusion criteria were applied: treatment for pituitary disorder (surgery and/or radiotherapy) at least 1 year before study entry, stable replacement therapy for other pituitary hormone deficiencies for at least 6 months (thyroid hormone deficiency, growth hormone deficiency, testosterone/estradiol deficiency, diabetes insipidus), age between 18 and 75 years and body weight between 50 and 100 kg.^14^ Main exclusion criteria were drug abuse or dependence, shift work, previous Cushing's disease, a history of frequent episodes of hypocortisolism and use of antiepileptic drugs.^14^

Patients treated with cortisone acetate were converted to treatment with hydrocortisone in a bioequivalent dose during a 4‐week run‐in period. The hydrocortisone dose during the run‐in period averaged 25 mg per day (0.31 mg/kg body weight). After the run‐in phase, patients were randomized to either first receive a lower hydrocortisone dose for 10 weeks followed by a higher hydrocortisone dose for 10 weeks, or vice versa. During the lower dose period, patients received 0.2‐0.3 mg per kg body weight per day (total daily doses ranged from 15 to 20 mg per day), taken before breakfast, before lunch and before dinner. During the higher dose period, patients received the double amount, 0.4‐0.6 mg per kg body per day (total daily doses ranged from 30 to 40 mg per day), taken at the same time points of the day. In case of intercurrent illness, doubling or tripling of the dose was allowed for a maximum of 7 successive days. Compliance with study medication was assessed as described.^14^ After each treatment period, blood sample drawing 1 hour after ingestion of the morning dose of hydrocortisone was done and 24‐hour urine collection was performed. The initial study population existed 47 patients that completed both study periods.^14^ For present analysis, samples of 19 patients were available.

#### 2.3.3 Exposure to and pharmacokinetics of hydrocortisone

In patients with secondary adrenal insufficiency, estimations of the individual pharmacokinetic parameters were calculated as described in detail elsewhere.^15^ In short, the Kinpop module of MwPharm version 3.81 was used to calculate a one‐compartment and two‐compartment population model for plasma total cortisol. The models were calculated using an iterative two‐stage Bayesian procedure and validated with the Monte Carlo analysis using MwPharm. The one‐compartment model showed the best fit, as measured with the relative root mean squared error which is a measure of precision, and was kept as the final model for further pharmacokinetic analysis. Individual pharmacokinetic parameter was calculated by maximum a posteriori Bayesian estimation using the noncompartment final model for plasma total cortisol for the two different doses of HC. Total body clearance (CL), volume of distribution (*V*_d_), elimination half‐life (*t*_1/2_) and area under the curve (AUC24h) were calculated.

It is not possible to determine (without the use of stable isotopes) whether cortisol in plasma is solely present as the consequence of hydrocortisone substitution or as a consequence of both residual endogenous cortisol production and substitution therapy. We therefore studied cortisol exposure and pharmacokinetics to estimate whether higher producers of 11‐deoxycortisol (suggestive of higher residual endogenous cortisol production) contributed to more cortisol exposure or a longer cortisol half‐life.

#### 2.3.4 Laboratory analysis

Cortisol (F), cortisone (E), 11‐deoxycortisol (11S), corticosterone (B) and 11‐deoxycorticosterone (11DOC) were measured by isotope dilution liquid chromatography tandem mass spectrometry (LC‐MS/MS). The measurement of these steroids in plasma was performed essentially as described by Hawley et al, using cortisol‐D4, E‐D7, 11S‐13C3, 21S‐D4, B‐D4, and 11DOC‐13C3 as internal standard.^16^ In short, 200 µL of plasma was used, and isotope labelled internal standards were added and incubated for 10 minutes. Samples were extracted using a 400 µL supported liquid extraction plate (Biotage). Samples were eluted with methyl tert‐butyl ether. The resulting eluate was evaporated and reconstituted in 200 µL 40% methanol‐water (v/v) and vortex‐mixed. 40 μL was injected on the online solid‐phase extraction system (Acquity Online SPE Manager, Waters), and the sample was extracted using a XBridge C18 cartridge (Waters). A Kinetex Biphenyl 100 × 2.1 mm column with a 2.6 μm particle size (Phenomenex) was used for chromatograpic separation. The mobile phase consisted of 2 mmol/L NH4Ac + 0.05% FA in 10% MeOH (A) and 2 mmol/L NH4Ac + 0.05% FA in 100% MeOH (B). Total runtime, including online extraction, was 9:00 minutes. Mass spectrometric (MS) detection was performed with a Xevo TQ‐s (Waters) operated in positive electrospray ionization and selective reaction monitoring mode. MS settings and transitions are shown in the supplement Table. The intra‐ and interassay coefficients of variation (CVs) of F, E, 11S, 21S, B and 11DOC were <3.3, <6.0, <4.1, <7.9, <3.9 and <7.6%, respectively. The limit of quantification (LOQ) for F, E, 11S, 21S, B and 11DOC was 0.2, 1.1, 0.025, 0.42, 0.17 and 0.024 pmol/L. Urinary free cortisol was also measured using isotope dilution LC‐MS/MS, as previously described.^17^

#### 2.3.5 Statistics

Data are presented as mean ± standard deviation or median (interquartile range) when appropriate. Normality of the data was checked using QQ‐plots and histograms. Parametric statistics were used between groups for most of the variables. However, differences in the pharmacokinetic parameters for plasma total cortisol and urinary free cortisol between groups separated by the median of 11‐deoxycortisol were assessed using the Mann‐Whitney *U* test. This test was separately used for both the lower and the higher dose of HC.

## 3 RESULTS

### 3.1 Subject characteristics

Twenty patients with PAI with matched controls and 19 with SAI were compared. Clinical characteristics are shown in Table 1. Age and age at diagnosis was not different between groups. The group of PAI consisted of more women than the SAI group (75% vs 53%). Weight, BMI and waist were all significantly higher in the SAI patients. Patients with PAI used a HC morning dose and total daily dose that were intermediate between the lower and higher HC dose of SAI patients. When corrected for body weight, the higher dose of hydrocortisone in SAI was similar to the dose of the PAI patients.

**Table 1:** Clinical characteristics of study patients

	Controls (n = 20)	Primary adrenal insufficiency patients (n = 20)	Secondary adrenal insufficiency patients (n = 19)	*P*‐Value a
Age (y)	54 ± 17	54 ± 17	52 ± 12	0.747
Sex (males/females), n	4/16	4/16	9/10	
Age at diagnosis (y)	na	40 ± 18	32 ± 19	0.169
Body weight (kg)	69.3 ± 13.5	69.7 ± 13.3	81.6 ± 14.7	0.012
BMI (kg/m^2^)	23.5 ± 3.4	24.7 ± 4.7	27.2 ± 5.1	0.132
Waist circumference (cm)	84.0 ± 11.5	86.9 ± 11.7	98.1 ± 11.0	0.004
Diagnosis		Autoimmune adrenalitis/other 19/1	Pituitary adenoma/congenital hormone deficiencies/cranial tumour at distant/craniopharyngioma/other 8/3/3/2/3	n/a
Hydrocortisone treatment at study^a^				
Total daily dose (mg/d)	‐	27.8 ± 7.0	Lower dose: 17.8 ± 2.3	
			Higher dose: 35.5 ± 4.7	
Morning dose (mg)	‐	16.0 ± 4.8	Lower dose: 9.1 ± 1.2	
			Higher dose: 18.2 ± 2.5	
Weight‐corrected daily morning dose (mg/kg BW)		0.23 ± 0.06	Lower dose: 0.11 ± 0.01	
			Higher dose: 0.23 ± 0.03	
Number of daily dosings, n = 1/2/3	‐	1/16/3	0/0/19	
Fludrocortisone dose (µg/d)	‐	75 ± 26	‐	
Additional hormone deficiencies			TSH/GH/LH, FSH/ADH 18/11/13/4	

Note

Data are mean ± standard deviation.

Abbreviations: BW, body weight; na, not applicable.

a P value of primary vs secondary adrenal insufficiency patients. Controls are matched to primary adrenal insufficiency patients.

### 3.2 11‐deoxycortisol, corticosterone and 11‐deoxycorticosterone

**Figure 1:**
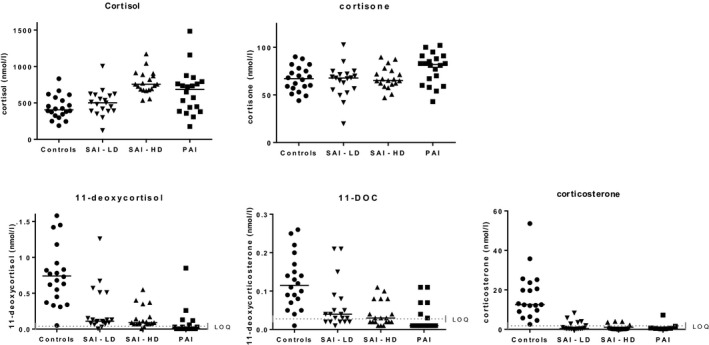
Limit of quantification (LOQ). Individual data and the median are plotted

Quantifiable amounts of 11‐deoxycortisol or corticosterone or 11‐deoxycorticosterone were present in 100% of controls, in 94.7% of patients with SAI and in 50% of patients with PAI. The individual percentages for each of these compounds for the study groups are given in Table 2. The individual concentrations for cortisol, cortisone and all above‐mentioned steroid precursors are shown in the Figure 1. Mean concentrations (SD) in controls for 11‐deoxycortisol or corticosterone or 11‐deoxycorticosterone were 0.78 (0.39) nmol/L, 17.0 (12.3) nmol/L and 0.13 (0.07) nmol/L. Increasingly lower concentrations of 11‐deoxycortisol, corticosterone and 11‐deoxycorticosterone were found in patients with SAI on a lower HC dose, SAI on a higher HC dose and PAI patients, respectively.

**Table 2:** Percentages of patients and controls with quantifiable amounts of 11‐deoxycortisol, 11‐deoxycorticosterone and corticosterone

	11‐deoxycortisol	11‐deoxycorticosterone	corticosterone
Controls (%)	100	95	95
PAI (%)	40	30	35
SAI on HC lower dose (%)	94.7	94.7	100
SAI on higher HC dose (%)	94.7	94.7	100

### 3.3 Exposure to and pharmacokinetics of hydrocortisone in SAI patients

Patients with SAI who had 11‐deoxycortisol concentrations below the median on either the lower dose or the higher dose of HC showed no differences in pharmacokinetic parameters of HC, especially not elimination half‐life (*t*_½_) and 24‐hour cortisol exposure in plasma (AUC 24h). In addition, urinary 24‐hour cortisol excretion was also not different in these patient groups (Table 3).

**Table 3:** Comparison of pharmacokinetics of oral immediate release hydrocortisone in patients below and above the median for 11‐deoxycortisol and total urinary free cortisol in 24‐h urine collection

LOWER DOSE HC	11 deoxycortisol Below median (N = 10)	11 deoxycortisol Above median (N = 9)	*P*‐Value
11‐deoxycortisol (nmol/L)	0.08 (0.05; 0.10)	0.51 (0.13; 0.62)	<0.001
CL (L/h) total cortisol	14.66 (8.89; 22.03)	10.94 (6.65; 14.13)	0.274
*V*_d_ (L) total cortisol	34.92 (25.50; 48.00)	35.89 (30.11; 45.10)	1.000
*t*_1/2_ (h) total cortisol	1.58 (1.43; 2.45)	2.25 (1.48; 4.16)	0.203
AUC24h (h*nmol/L)	3166.16 (2400.11; 5697.60)	4593.85 (3218.45; 6995.72)	0.237
24 h UFC (nmol)	48.50 (28.75; 129.35)	98.00 (77.25; 113.50)	0.274
HIGHER DOSE HC			
11‐deoxycortisol (nmol/L)	0.08 (0.06; 0.09)	0.17 (0.08; 0.39)	0.010
CL (L/h) total cortisol	12.26 (8.81; 19.36)	15.95 (12.53; 20.72)	0.278
*V*_d_ (L) total cortisol	50.33 (33.55; 55.62)	48.45 (44.52; 58.82)	0.549
*t*_1/2_ (h) total cortisol	2.20 (1.78; 3.73)	2.01 (1.88; 2.60)	0.842
AUC24h (h*nmol/L)	7282.13 (5501.77; 11119.30)	5762.56 (4945.16; 6795.66)	0.211
24 h UFC (nmol)	301.50 (191.25; 530.75)	274.50 (205.25; 361.75)	0.573

Note

Data are median (IQR).

Abbreviations: 24 h UFC: urinary free cortisol in 24 hour; AUC24h, area under the curve of hydrocortisone are shown; CL, The pharmacokinetic parameters clearance; *t*_1/2_, elimination half‐life; *V*_d_, volume of distribution.

## 4 DISCUSSION

This study demonstrates that adrenal cortisol production is likely to continue in almost all patients with SAI. Interestingly, even in half of the PAI patients, precursors of cortisol and aldosterone were quantifiable. Remarkable is that all patients had long‐standing disease on average 15‐20 years. Our study points to interesting novelties in physiology both in PAI and SAI. In PAI patients, the presumed autoimmune destruction of the adrenal gland may not be complete in a substantial number of patients who show residual functioning of adrenal cortical tissue, albeit at a very low level. And in SAI patients, the adrenal glands show evidence of cortisol production, even after long‐standing absence of significant amounts of ACTH and in the presence of negative feedback provided by exogenous hydrocortisone. Based on the duration of the pituitary disease, which was between 10 and 20 years in our patients, this is a new finding. It is well‐known that duration of more than 30 days after onset of ACTH deficiency leads to adrenal atrophy.^18^ However, results of our study indicate that this functional atrophy appears not to be complete in long‐standing disease. Altogether, our results show that some residual functioning of the adrenal cortical gland is common in adrenal insufficiency, especially in secondary adrenal insufficiency.

Residual adrenocortical functioning during hydrocortisone is not frequently studied during substitution therapy. Usually a strategy is chosen to temporarily withdraw hydrocortisone treatment followed by measurement of an early morning cortisol or by dynamic testing,^8^ but this is usually done to confirm or reject the diagnosis of adrenal insufficiency preferentially at an early stage after disease onset.^19^

Hydrocortisone withdrawal is possible and may certainly be of value. However, by this approach we obviate the need for withdrawal. It opens the possibility to routinely map the underlying function of the adrenal cortex in patients on hydrocortisone substitution at any moment visiting the endocrine outpatient clinic. In addition, withdrawal of hydrocortisone is not clinical practice during long‐term therapy, as is the case in our patient group who were on approximately 10‐20 years after their diagnosis of adrenal insufficiency.

Techniques involving stable isotopes may give insight in patients with adrenal insufficiency, but these studies have not been performed with the focus on residual endogenous cortisol production. In addition, this technique is not a tool for daily clinical practice and only feasible in specialized laboratories. In contrast, plasma steroid profiling by means of LC‐MS/MS is a newly developed technique available since a few years and has been used for the evaluation of secreting adrenal adenomas.^20^, ^21^, ^22^ Mass spectrometry is an efficient and specific utility superior in this context to the cumbersome immuno‐assays and without disturbing cross‐reactivity.^21^, ^23^ We developed a LC‐MS/MS that combines a low LOQ for all measured steroids (in the low picomolar range) and short retention time (Table S1), making it possible to measure multiple samples in a short period of time. Another strength of the present study is the pharmacokinetic analysis under two HC dose conditions. In addition, urinary free cortisol was taken into account. Thus, several markers of cortisol exposure were available to assess if residual endogenous cortisol production adds to total cortisol exposure in these patients.

Evidence of production of cortisol precursors in adrenal insufficiency may have interesting implications. For example, low concentrations of 11‐deoxycortisol or corticosterone may affect quality of life or cognition. In agreement with this, Nixon and co‐workers have shown that corticosterone rather than cortisol may be the primary glucocorticoid for the brain, as a consequence of tissue‐specific expression of transmembrane exporters of corticosteroids.^24^ In addition, Schweitzer found that 11‐deoxycortisol to cortisol ratios were different in depressed patients when compared to normal volunteers.^25^

The use of 11‐deoxycortisol may be of potential interest in several areas. Firstly, as illustrated in this study, as indicator of residual adrenal cortisol production. This has potential in patients with hydrocortisone tapering regimes to assess recovery of adrenal function or in patients after pituitary surgery who fail to show an adequate basal or stimulated cortisol and are often put on lifelong hydrocortisone replacement therapy without additional testing. Secondly, 11‐deoxycortisol may be an indicator (biomarker) of oversubstitution with hydrocortisone in SAI. Current practice is to make this assessment on clinical grounds, but well‐defined criteria are lacking. In fact, due to the complexity of the entire HPA axis physiology with its interindividual differences in glucocorticoid sensitivity personalized treatment doses are necessary. The search of biomarkers to assess adequacy of hydrocortisone substitution is therefore ongoing. The use of 11‐deoxycortisol as indicator of oversubstitution is new, and prospective data sampled under different doses hydrocortisone and related to side effects of glucocorticoids, such as hypertension, dyslipidemia, diabetes and osteoporosis, must be performed to address this. Thirdly, cortisol is produced directly by the adrenals but also by regeneration of cortisone by 11‐HSD1 activity. Differentiation between these two pathways is only possible today by enzymatic inhibition or by use of stable isotopes.^26^ The use of 11‐deoxycortisol may be of help here to assess the relative contribution of direct cortisol production by the adrenal glands.

Several shortcomings need to be addressed. First, this is a pilot study addressing feasibility and potential implications in a relatively small study of 20 PAI with controls and 19 SAI patients. The data presented here are an ancillary analysis with samples derived from two different studies. Both study protocols, although well defined, were therefore slightly different, and this may have resulted in differences in timing of blood sampling. However, both PAI patients and SAI patients with the higher dose received similar amounts of HC as calculated by mg per kilogram body weight, and indeed, comparable levels of cortisol were measured. Future studies should be specifically designed to address this topic. Secondly, we added a group of PAI patients with the initial assumption that these were complete negative controls, thus establishing that no extra‐adrenal production of cortisol precursors was present. Indeed, this was the case in many patients with PAI, confirming that the enzymatic machinery to produce cortisol is only present in the adrenal glands. However, much to our surprise, half of the patients with autoimmune primary adrenal insufficiency showed some evidence of adrenal cortical functioning. Additional testing is necessary to confirm this and gain further insight. Thirdly, the inclusion of dynamic testing of adrenal cortex hormone secretion—such as Synacthen test or insulin tolerance test—would have introduced a gold standard reference test for assessing residual cortisol secretion. Therefore, the finding of a correlation between the level of cortisol precursor 11‐deoxycortisol, aldosterone precursors and the basal/stimulated cortisol secretion would have strengthened the value of these precursors as biomarkers of adrenal cortex residual secretion in AI. Lastly, because of this study design, we lack data of the plasma/urine cortisol concentrations of PAI patients.

We conclude that cortisol synthesis is ongoing in SAI patients based on concentrations of 11‐deoxycortisol, albeit at very low levels because no increase total cortisol exposure was observed in SAI patients with higher 11‐deoxycortisol concentrations. This seems plausible, but additional data validated against other methods in healthy volunteers and patients are needed to gain further insight in this. However, when looking at the absolute concentrations of 11‐deoxycortisol, which appears to be <1/1000th of the normal cortisol concentrations at 9 am, one comes to similar conclusions. In conclusion, adrenal corticosteroid production is likely to continue during treatment in a considerable percentage of patients with PAI and in almost all patients with SAI. In patient with SAI, the higher doses of HC suppressed 11‐deoxycortisol concentration, suggesting feedback inhibition of endogenous cortisol production. Whether residual cortisol production in adrenal insufficiency has clinical consequences remains to be determined.

## CONFLICT OF INTEREST

The authors declare no conflicts of interest.

## AUTHOR CONTRIBUTIONS

Annet Vulto and Martijn van Faassen: have made substantial contributions to conception and design, or acquisition of data, or analysis and interpretation of data; Annet Vulto, Ragnhildur Bergthorsdottir, Martijn van Faassen, Ido P Kema, Gudmundur Johannsson and André P van Beek: been involved in drafting the manuscript or revising it critically for important intellectual content; Each author given final approval of the version to be published. Each author should have participated sufficiently in the work to take public responsibility for appropriate portions of the content; Annet Vulto, Ragnhildur Bergthorsdottir, Martijn van Faassen, Ido P Kema, Gudmundur Johannsson and André P van Beek: agreed to be accountable for all aspects of the work in ensuring that questions related to the accuracy or integrity of any part of the work are appropriately investigated and resolved.

## Data Availability

The data that support the findings of this study are available from the corresponding author upon reasonable request.
