# Supplementary material for: Residual endogenous corticosteroid production in patients with adrenal insufficiency
Source: Clin Endocrinol (Oxf). 2019 Jun 20;91(3):383–90. doi: 10.1111/cen.14006 (PMC6851705; doi:10.1111/cen.14006)
Supplement: Supplementary file 1 [file CEN-91-383-s001.docx]

**Supplemental Table. MS settings and transitions for each respective analyte.**

| **Analytes** | **Precursor (m/z)** | **Product (m/z)** | **Cone voltage (V)** | **Collision energy (eV)** |
| --- | --- | --- | --- | --- |
| 11DOC (QN) | 331.25 | 97.15 | 30 | 22 |
| 11DOC (QL) | 331.25 | 109.15 | 30 | 25 |
| 11DOC-13C3 (QN) | 334.25 | 100.15 | 30 | 22 |
| 11DOC-13C3 (QL) | 334.25 | 112.15 | 30 | 25 |
| 11S (QN) | 347.35 | 97.15 | 30 | 23 |
| 11S (QL) | 347.35 | 109.15 | 30 | 28 |
| 11S-13C3 (QN) | 350.35 | 100.10 | 30 | 23 |
| 11S-13C3 (QL) | 350.35 | 112.10 | 30 | 28 |
| Cortisol (QN) | 363.30 | 121.10 | 30 | 46 |
| Cortsol (QL) | 363.30 | 326.90 | 30 | 39 |
| Cortisol-D4 (QN) | 367.30 | 121.10 | 30 | 46 |
| Cortisol-D4 (QL) | 367.30 | 330.90 | 30 | 39 |
| B (QN) | 347.35 | 121.00 | 30 | 12 |
| B (QL) | 347.35 | 293.35 | 30 | 15 |
| B-D4 (QN) | 351.35 | 121.00 | 30 | 12 |
| B-D4 (QL) | 351.35 | 297.35 | 30 | 15 |
| Cortisone (QN) | 361.30 | 121.10 | 30 | 35 |
| CortisoneE (QL) | 361.30 | 163.10 | 30 | 31 |
| Cortisone-D7 (QN) | 368.30 | 125.10 | 30 | 35 |
| Cortisone-D7 (QL) | 368.30 | 169.10 | 30 | 31 |
| 21S (QN) | 347.25 | 121.15 | 30 | 25 |
| 21S (QL) | 347.25 | 311.20 | 30 | 15 |
| 21S-D4 (QN) | 351.35 | 121.15 | 30 | 25 |
| 21S-D4 (QL) | 351.35 | 315.20 | 30 | 15 |

Abbreviations: 11DOC, 11-deoxycorticosterone ; 11S, 11-deoxycortisol ; 21S, 21-deoxycortisol ; QL, qualifier; QN, quantifier.
